# Determination of oxytetracycline residues in cattle meat marketed in the Kilosa district, Tanzania

**DOI:** 10.4102/ojvr.v82i1.911

**Published:** 2015-11-27

**Authors:** Zuhura I. Kimera, Robinson H. Mdegela, Consolatha J.N. Mhaiki, Esron D. Karimuribo, Faith Mabiki, Hezron E. Nonga, James Mwesongo

**Affiliations:** 1 Department of Fisheries Development, Ministry of Livestock and Fisheries Development, Tanzania; 2 Department of Veterinary Medicine and Public Health, Sokoine University of Agriculture, Tanzania; 3 Department of Soil Science, Sokoine University of Agriculture, Tanzania; 4 Department of Physical Science, Sokoine University of Agriculture, Tanzania

## Abstract

Oxytetracycline is used to treat various diseases in cattle. However, its use may be associated with unacceptable residue levels in food. Oxytetracycline residues in tissues from indigenous cattle were determined in a cross-sectional study conducted in the Kilosa district, Tanzania, between November 2012 and April 2013. A total of 60 tissue samples, including muscle, liver and kidney, were collected from slaughterhouses and butchers and analysed for oxytetracycline using high-performance liquid chromatography. Oxytetracycline residues were found in 71.1% of the samples, of which 68.3% were above acceptable regulatory levels. The mean concentration of oxytetracycline across tissues was 3401.1 µg/kg ± 879.3 µg/kg; concentrations in muscle, liver and kidney were 2604.1 µg/kg ± 703.7 µg/kg, 3434.4 µg/kg ± 606.4 µg/kg and 3533.1 µg/kg ± 803.6 µg/kg, respectively. High levels of oxytetracycline residue in meat from indigenous cattle may pose a health threat to consumers in Kilosa. The findings possibly reflect a general lack of implementation of recommended withdrawal periods, ignorance about drug use and lack of extension services. Strict regulation of the use of antimicrobial drugs in the livestock industry and associated testing of animal-derived food sources prior to marketing are required.

## Introduction

In Tanzania, livestock farming generally follows traditional practices whereby pastoralists and agro-pastoralists rear mostly local breeds for meat and milk production (Ministry of Livestock and Fisheries Development 2010). Livestock farming faces several constraints, including diseases, poor genetic potential of animals, poor management and nutrition, and drought (Mellau, Nonga & Karimuribo 2010). Owing to limited extension services and poor animal health delivery systems, farmers buy veterinary drugs from veterinary shops and treat their livestock themselves. However, when drugs are administered by non-professionals, correct dosages and withdrawal periods are unlikely to be observed, which poses a potential hazard to human health (Barton 2000). The possible effects include toxic or allergic reactions, development of bacterial resistance and disturbance of normal intestinal microflora composition (Abbasi *et al*. 2011; Pena *et al*. 2005; Uekane, Neto & Gomes 2011).

The use of antimicrobial agents in food-producing animals has become a notable public health concern, especially in developing countries where such drugs are administered indiscriminately (Bedada & Zewde 2012; Muriuki *et al*. 2001; Olatoye & Basiru 2013). The increased use of antimicrobials in animal production is due to their being applied both therapeutically and prophylactically; some are also routinely added to animal feeds at sub-therapeutic levels for growth promotion (Bedada & Zewde 2012; Nonga *et al*. 2010).

Oxytetracycline is one of the most commonly used antibiotics in livestock production in Tanzania and other African countries (Katakweba *et al*. 2012; Olufemi & Agboola 2009). Apart from being a broad-spectrum antibiotic, oxytetracycline is also cheap, readily available from veterinary shops and accessed easily, without restrictions, by farmers (Nonga *et al*. 2009; Olatoye & Basiru 2013). Katakweba *et al*. (2012) reported that a number of drugs, such as oxytetracycline, are used abusively to treat and protect cattle against various diseases. Moreover, informal vendors are often seen selling oxytetracycline and other tetracycline-based drugs at informal markets and along the road, without any prescription being required or restrictions imposed (Bedada & Zewde 2012; Karimuribo *et al*. 2013).

To protect humans from harmful effects of veterinary drug residues in animal-derived food sources, the United Nations Food and Agriculture Organization (FAO) and the World Health Organization (WHO) have set standards for maximum residue limits in foods. These limits apply to the parent drug or chemical and its metabolites that may accumulate and be deposited or stored within the cells, tissues or organs following administration of the compound. The acceptable maximum residue limits for tetracycline-based compounds, including chlortetracycline and oxytetracycline, are set at 200 µg/kg, 600 µg/kg and 1200 µg/kg for cattle-derived muscle, liver and kidney, respectively.

Withdrawal periods of 5–20 days are recommended before slaughter, depending on the species and the nature of the food products (Blanchflower *et al*. 1997). However, regulatory bodies in Tanzania have not yet set withdrawal periods for veterinary drugs and farmers rely only on the directions given in the package insert. This information is always written in English, a language the majority of Tanzanian farmers do not understand. Therefore, farmers rarely comply with the recommendations and usage is also not monitored by the responsible regulatory authorities. Consequently, veterinary drug residues are likely to be present in food of animal origin. This study sought to investigate the presence of excessive concentrations of oxytetracycline residues in cattle meat marketed in the Kilosa district, Tanzania.

## Materials and methods

### Sample collection

This study was conducted in the Kilosa district (5°55’–7°53'S, 36°30’–37°30'E), which is located approximately 300 km west of Dar es Salaam in east central Tanzania. The district is divided into three zones, namely Kilosa, Gairo and Mikumi, and spans a land area of 19 056 km^2^. The human population of the district is documented as 438 175 (National Bureau of Statistics 2012).

Samples were obtained from cattle slaughtered at the Kilosa, Gairo and Mikumi slaughter slabs and at the Parakuyo and Chakwale livestock markets. Records obtained during sampling indicated that the animals originated from ten different villages across the district.

Animals were selected for sampling using simple random sampling techniques. Information such as the name of the owner or supplier, the village of origin, any pathological lesions at the time of sampling and inspection status prior to slaughter was collected before sampling. Three samples (muscle, liver and kidney) of 100 g – 200 g each were obtained from each of 20 animals, yielding 60 tissue samples in total. All samples were collected in separate polythene bags and transported on ice to the analytical laboratory at the Sokoine University of Agriculture (Faculty of Veterinary Medicine). The samples were stored in a freezer at −20 °C for approximately 1 week and thawed at room temperature for eight hours before analysis.

### Sample analysis

High-performance liquid chromatography (HPLC) was used to analyse the samples. All the reagents and chemicals were of HPLC or analytical grade. Reagents included oxytetracycline standard (Sigma, St Louis), oxalic acid dihydrate, citric acid monohydrate and disodium ethylenediaminetetraacetate (Na_2_EDTA) (Techno Pharmachem, India), anhydrous disodium hydrogen phosphate (Carlo Ebra, Milan), methanol, acetonitrile and HPLC water (Carlo Ebra, Milan). Whatman membranes, microsyringe membrane filters (Chromafil CA 20/25s), nylon membranes (P/N 0235-0301) and Cronus C-18 solid-phase extraction cartridges (200 mg/3 mL, Labhut) were used for the chromatography steps.

Each tissue sample (5 g) was homogenised three times in a Mcllvaine buffer–EDTA solution (20 mL, 20 mL and 10 mL) and collected in a 50-mL polypropylene centrifuge tube. The mixture was then centrifuged at 4000 g for 10 min and the supernatant was filtered through a single Whatman filter, pre-moistened with 2 mL Mcllvaine buffer–EDTA solution, into a 250-mL sidearm flask. The solid-phase extraction was conducted by conditioning the extraction cartridge with 20 mL methanol followed by 20 mL HPLC-grade water. The final sample extract was applied to an 18-carbon cartridge, which was subsequently washed with 20 mL HPLC-grade water. Oxytetracycline was eluted with 6 mL methanolic oxalic acid solution into a 10-mL volumetric flask, which was then filled with water to volume.

Muscle, liver and kidney sample extracts were analysed for oxytetracycline residues according to AOAC Official Method 995.09 (AOAC International 2000), with some modifications. The HPLC instrument (Shimadzu 20AD) was fitted with an autosampler (SIL-20 AHT) and a UV detector at 350 nm was used for analysis. A reversed-phase 18-carbon column (150 mm × 4.60 mm; particle size, 5 µm; Supelco) was used at 25 °C for separation. The sample injection volume was 1 µL at a flow rate of 0.8 mL/min. A low-pressure gradient system, consisting of water and methanol, acetonitrile and aqueous oxalic acid (10:30:60) as the mobile phase, was applied for a retention time of 15 min. To determine residues in the samples, they were analysed concurrently with the oxtetracycline standard solutions (0.05 µg/mL, 0.1 µg/mL, 0.25 µg/mL, 0.5 µg/mL and 1.0 µg/mL). The extract from each sample was injected in duplicate to obtain an average peak height of positive samples. Samples were considered positive for oxytetracycline residue if their retention time and peak corresponded to that of the reference standards. The retention time of the reference standard was 4.3 min.

Oxytetracycline residues in tissue sample extracts were quantified against the aforementioned concentrations of the oxytetracycline reference standards. The standards were analysed in duplicate and the peak areas appropriate to specific standard concentrations were measured. These were used to calculate the residue concentrations in sample extracts.

### Control samples

As it was not possible to obtain cattle that had not been treated with oxytetracycline or other veterinary drugs prior to slaughter, three oxytetracycline-free guinea pigs (all of the same age, sex and weight) raised at the Faculty of Veterinary Medicine, Sokoine University of Agriculture, were used as controls. One guinea pig served as negative control, whereas the other two were injected with 20% oxytetracycline (Laprovet, Indre-et-Loire) at 10 mg/kg body weight and 20 mg/kg body weight, respectively. After 24 h the three control animals were placed in a gas chamber (61.4% CO_2_, 20.3% O_2_ and 18.29% N_2_) for 5 min before being humanely killed. Muscle, liver and kidney tissue samples (5 g each) were subjected separately to the extraction, clean-up and elution procedures as described for the test samples derived from cattle. Two samples of each of the three tissue types were taken from each of the control animals. The control samples were run through the HPLC column under similar conditions to the oxytetracycline standard solutions. A blank sample eluted from the solid-phase extraction cartridge was included to check for the analytical column efficiency during extraction.

Operational conditions of the HPLC instrument were tested to ensure the robustness of the method. These checks included varying the percentage of solvents in the mobile phase (methanol: 10% – 30%; acetonitrile: 10% – 30%; oxalic acid: 50% – 70%), the column temperature (from 25 °C to 40 °C)and the flow rate (from 0.6 mL/min to 1 mL/min). The pH of the buffer was changed from 2.0 to 7.0. Stability of the sample and standard solutions at room temperature was also tested.

### Data analysis

The data were analysed using Epi Info (version 7) (Centre for Disease Control, Atlanta, USA). The Chi-square statistic and confidence intervals were used to compare proportions; a probability of *P* < 0.5 was considered statistically significant. Descriptive statistics were used to compute means, standard deviations and range. Analysis of variance (ANOVA) was used to compare differences in means of continuous variables.

## Ethical considerations

Permission for this study was granted by the Executive Directors of the Kilosa District Council and ethical approval for the study was obtained from the Ethical Committee of the Sokoine University of Agriculture. The university issued a research permit letter on behalf of the Tanzanian Commission for Science and Technology.

## Results

### Control samples

**FIGURE 1:**
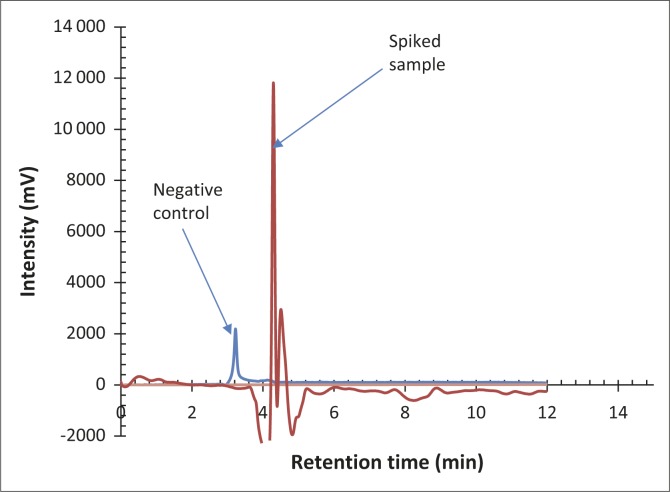
Combined chromatogram of the negative control sample (without oxytetracycline) and the sample spiked with 10 mg/kg 20% oxytetracycline.

**FIGURE 2:**
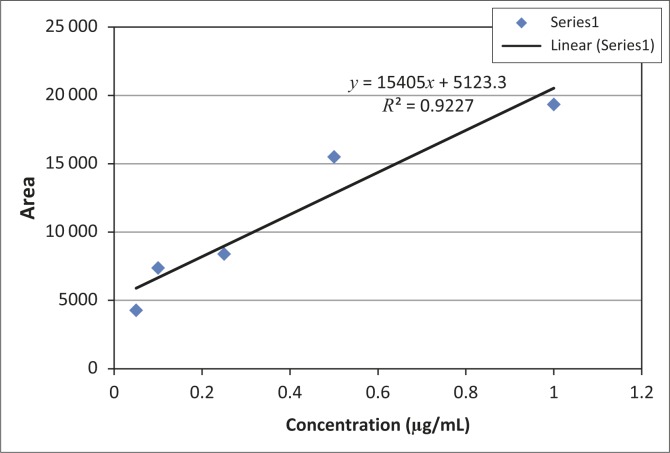
Calibration curve depicting the best-fit line from analytical oxytetracycline standards.

The non-spiked samples from the control animals peaked at a different time from that of the analytical standards, whereas both oxytetracycline-spiked samples peaked at 4.3 min as expected. The higher concentration of oxytetracycline was associated with a higher peak. The peak for extracted oxytetracycline was also detected at 4.3 min and was of similar height to the spiked control sample. Between 79% and 83% oxytetracycline was recovered from the samples. The limit of detection and the limit of quantification were 1.936 mg/kg and 6.7 mg/kg, respectively. The correlation coefficients associated with the linear regression for the analytical oxytetracycline standard (Figure 1) and test samples (Figure 2) were *R*^2^ = 0.92 and *R*^2^ = 0.94, respectively. (The linear regression equation is shown in Figure 2). For quantification purposes, the best-fit line was expected to be 99%, but based on the local environment, nature of the equipment and the laboratory used, the obtained fits were considered adequate.

Of the 20 animals sampled, 17 (85%) tested positive for oxytetracycline residues. Moreover, 68.3% of the positive tissue samples contained oxytetracycline residues above the acceptable levels for muscle, liver and kidney (Food and Agriculture Organization/World Health Organization 2014). The mean concentration of oxytetracycline residues across all tissues was 3401.1 µg/kg ± 879.3 µg/kg. For the respective tissue types, the mean concentrations were 2604.1 µg/kg ± 703.7 µg/kg for muscle, 3434.4 µg/kg ± 606.4 µg/kg for liver and 3533.1 µg/kg ± 803.6 µg/kg for kidney tissue. Oxytetracycline concentrations were higher than the acceptable levels in all tissue types sampled from animals from the Kilosa and Mikumi zones (Table 1). Lower concentrations of oxytetracycline were found in samples collected from cattle slaughtered in the Gairo zone (Table 1).

**TABLE 1:** Oxytetracycline concentration in cattle tissue from the Kilosa district, Tanzania.

Zone of sample collection	Number of samples (*n*)	Antibiotic concentrations (g/kg)				
		Kidney	Liver	Muscle
Kilosa	21	1548.0 ± 708.3	1484.8 ± 493.2	1126.7 ± 204.3
Mikumi	18	1553.2 ± 957.1	1307.6 ± 660.1	1083.3 ± 282.7
Gairo	21	388.3 ± 183.5	582.4 ± 220.6	357.8 ± 192.8

## Discussion

The purpose of this study was to determine the presence of oxytetracycline residues in beef from indigenous cattle in the Kilosa district, Tanzania. The results showed a high residual presence of oxytetracycline (71.1%), with a notable number of positive samples being above the acceptable maximum residue levels recommended for meat by the WHO and FAO. The finding can probably be attributed to widespread use of oxytetracycline for treatment and prevention of cattle diseases and is possibly exacerbated by failure to observe withdrawal periods (Karimuribo *et al*. 2013). Higher levels of oxytetracycline residue were found in liver and kidney tissue than in muscle, which can be attributed to their being organs of metabolism and excretion and therefore they are at greater risk of exposure to residues (Olatoye & Ehinmowo 2010). After administration, oxytetracycline enters all tissues and body fluids, but higher concentrations are found in the kidney, liver, bile, lungs and bones (Aiello & Moses 2010). Oxytetracycline is excreted mainly via urine and bile, which explains the high concentrations of residue observed in kidney and liver tissues in this study. As liver and kidney are considered luscious offal from cattle and are popular amongst most meat consumers in Tanzania, the detection of high levels of oxytetracycline residues in these tissues is of importance to public health.

The proportions of oxytetracycline-positive samples found in this study were higher than reported in other studies (Olufemi & Agboola 2009), although Bedada and Zewde (2012) reported a comparable proportion of oxytetracycline-positive muscle tissue samples (71.3%; *n* = 384) from cattle in Ethiopia. However, in a study analysing muscle tissue from cattle in the Morogoro and Dodoma municipalities, Tanzania, only 41.2% of samples tested positive for oxytetracycline residues (Mmbando 2004). Oxytetracycline residues in muscle tissue were reported in 45.6% and 54.4% of samples in studies from Kenya (Muriuki *et al*. 2001) and Nigeria (Olufemi & Agboola 2009), respectively, which are both relatively low compared to levels seen in the current study. This difference may be due to different sample types, laboratory methods and possible variation in oxytetracycline use depending on the animal management system of the locality.

Nisha (2008) reported that indiscriminate use of antibiotics to treat pyrexia, inflammation, wounds and viral diseases is associated with high levels of residues in edible tissues of food-producing animals. The high incidence of oxytetracycline residues observed in the current study probably reflects cattle being sold for slaughter whilst under a therapeutic or prophylactic regimen of oxytetracycline or animals being slaughtered before the end of the withdrawal period (5–7 days when the antibiotic has been administered at a dose of 10 mg/kg for 7 days [Aiello & Moses 2010]). It is also of concern that these levels of oxytetracycline were found in tissue from indigenous cattle, because more than 98% of the cattle population in Tanzania (approximately 21 million) are indigenous breeds and the main source of meat consumed in Tanzania (National Bureau of Statistics 2012). In addition, there is no official monitoring programme and consumer response towards the dangers posed by drug residues is passive. Thus, there is a risk of sustained consumer exposure to antibiotic residues and the associated effects on human health.

The high levels of antibiotic residues found may be due to insufficient knowledge about drug use and the lack of extension services. Livestock keepers in Kilosa are mostly Maasai, Mang'ati or Sukuma, who are known pastoralists in Tanzania, and livestock extension officers’ access to these farmers is problematic. There are few livestock field officers available and the majority are found in the vicinity of town centres such as Gairo. In the Kilosa and Mikumi zones, no veterinary services were offered because of the areas’ remoteness and poor infrastructure. The higher oxytetracycline residue levels found in samples from villages in the latter zones may be due to the lack of veterinary services, including extension services. The role of livestock extension officers is to advise farmers on proper animal management systems and disease control programmes, including vaccination. Our findings support the conclusion of Muriuki *et al*. (2001) that variation in residue levels – even from the same district – reflects the variation in animal husbandry practices as used by different livestock keepers and in different areas.

Easy access to antibiotics such as oxytetracycline, together with a lack of awareness, insufficient extension activities and inadequate usage guidelines from manufacturers, may lead to misuse and overuse of the drug and possibly failure to observe withdrawal periods. These actions may contribute to the presence of high levels of antibiotic residues in meat (Nisha 2008).

The lack of farmers’ awareness of the possible side-effects of antimicrobials and other drugs in humans also has to be considered (Karimuribo *et al*. 2013). Administration of drugs to food-producing animals requires consideration not only of effects on the animal but also of effects in humans who consume food from these animals. The high levels of antibiotic residues found in this study suggest that the public consuming animal products originating from the Kilosa district may have been exposed to antimicrobial residues. Our results, together with those of Mmbando (2004) and Nonga *et al*. (2009) about antibiotic residues in broiler chickens, suggest that some communities are exposed to small doses of antimicrobials from various animal food sources. This practice may contribute to the development of microbial resistance.

## Conclusion

The findings of our study may be indicative of the inappropriate use and management of veterinary drugs by livestock keepers in the Kilosa district specifically, but also more generally in Tanzania. We therefore recommend stricter regulation of the use of veterinary drugs in the livestock industry as well as the inspection of livestock products prior to marketing. Furthermore, livestock keepers need to be educated on the importance of adhering to the recommended drug withdrawal periods and possible human health effects associated with presence of veterinary drug residues in food of animal origin. Veterinarians and livestock officers should also promote alternative management options aimed at good animal husbandry and disease control measures.
